# Advancing fishery dependent and independent habitat assessments using automated image analysis: A fisheries management agency case study

**DOI:** 10.1371/journal.pone.0329409

**Published:** 2025-08-06

**Authors:** Scott N. Evans, Bronson Philippa, Carlo Mattone, Nick Konzewitsch, Renae K. Hovey, Marcus Sheaves, Gary A. Kendrick, Lynda M. Bellchambers

**Affiliations:** 1 Western Australian Fisheries and Marine Research Laboratories, Department of Primary Industries and Regional Development, Government of Western Australia, Hillarys, Western Australia, Australia; 2 School of Biological Sciences and UWA Oceans Institute, University of Western Australia, Crawley, Western Australia, Australia; 3 Marine Data Technology Hub, College of Science and Engineering, James Cook University, Townsville, Queensland, Australia; Central Marine Fisheries Research Institute, INDIA

## Abstract

Advances in artificial intelligence and machine learning have revolutionised data analysis, including in the field of marine and fisheries sciences. However, many fisheries agencies manage sensitive or proprietary data that cannot be shared externally, which can limit the adoption of externally hosted artificial intelligence platforms. In this study, we develop and evaluate two residual network-based automatic image annotation models to process fishery specific habitat data to support ecosystem-based fisheries management in the Exmouth Gulf Prawn Managed Fishery in Western Australia. Using an extensive dataset of 13,128 manually annotated benthic habitat images, we train a grid-based annotation model and an image-level object detection model. Both models demonstrated high overall accuracy, with the grid-based model achieving 90.8% and the image-level model 92.9%. Patch-wise accuracy of the image-level model was 74.2%, highlighting its ability to classify broader spatial context without requiring point-based labelling. Precision and recall values for both models often exceeded 70% for dominant habitat classes such as unconsolidated substrate, macroalgae, and seagrass. The development of these models supports the potential for cost-effective, robust, and scalable in-house habitat classification for fishery or ecoregion specific habitat data to support timely decision-making. Further, the grid-based model uniquely integrates spatial precision with compatibility to existing manual data workflows, enabling seamless adoption within many existing fisheries monitoring programs. Despite limitations, such as a class imbalanced dataset, both models present a scalable, data secure solution for fisheries management agencies. This study establishes a foundation for integrating artificial intelligence driven image analysis of proprietary fisheries data, to further support responsive, standardised and data-informed decision making.

## Introduction

The growth of artificial intelligence and machine learning algorithms has revolutionised scientific and industrial fields [1,2]. Innovative approaches to data analysis and interpretation [2,3] are removing historical bottlenecks to analyses [4–6] and producing results that can outperform human experts on benchmark datasets [6,7]. Automated Image Analysis (AIA) is a field of computer vision, artificial intelligence and machine learning that uses models or algorithms to label images based on their visual content or to identify similarities between image features and contextual information, with high efficiency and low subjectivity [8]. AIA has received substantial attention in recent times due to advances and affordability in video and photogrammetry technology increasing the capacity to capture and store high resolution digital imagery, which in turn has led to an increased level of image data to annotate [8–10]. The growth of AIA has been further driven by advances in computational technology, enabling greater integration with deep learning techniques such as convolutional neural networks (CNNs), which are highly effective at extracting subtle and complex features from imagery and supporting precise, granular habitat categorisation [11]. However, the success and accuracy of AIA models are dependent on the availability of large collections of expertly annotated training data, which are often associated with significant costs, at least initially, in resourcing [12–14].

For fisheries and marine science, AIA has demonstrated its ability to streamline data processing, thereby enhancing efficiency in scientific data provision that underpins sustainable management [4,15–20]. Identifying key fish and marine habitats like seagrass, macroalgae, and reef structures from images has traditionally been slow, manual, error prone, and resource intensive [21]. However, in recent years, the field has progressed from annotation tools that are dependent on manual input from human experts to analyse fish and marine habitats [22], such as Coral Point Count with excel extension [23], BIIGLE [24], TransectMeasure (www.seagis.com,au), EventMeasure (www.seagis.com.au), and VidSync (www.vidsync.org). This progression has been led by the development of software that utilises AIA for machine-assisted processing of benthic imagery, such as CoralNet [25,26] ReefCloud [27] and marine learning assisted image annotation in BIIGLE [6]. This type of development and use of AIA models represents a significant shift in practice, aiming to retain the accuracy of analysis while improving the scalability and speed of image annotation, ultimately reducing labour costs and expediting workflows.

The adoption of AIA for processing benthic imagery is an opportunity to meet the growing demand for marine habitat assessments, mapping and monitoring which provide rapid, robust ecological data to support both ecosystem-based fishery management (EBFM). EBFM is a holistic approach to fisheries management that considers the broad range of ecological, social and economic aspects of fisheries [28], and broader ecosystem-based management (EBM), including marine spatial planning [29–32]. However, for many government and fisheries management organisations, the use of externally hosted AIA platforms, such as ReefCloud or CoralNet, requires careful consideration of the confidentiality requirements of data collected by, or in collaboration with, stakeholders such as commercial fishers [33–35]. Despite the privacy, management, and permission settings offered by many external hosted platforms, there is a need for fisheries management agencies to take a precautionary approach due to potential legal liability and the real, or perceived, reputational risks associated with data breaches or unintentional exposure of proprietary information [36,37].

In addition, some externally hosted platforms remain static in nature, with limited flexibility for the user to directly access or adjust the code, or review the image databases that inform the model, to ensure standardisation with any new imagery, to meet the specific requirements of their study area [19,38].To address these challenges, especially where fishers or fisheries data cannot be aggregated and anonymised before external upload [36], fisheries agencies need the ability to develop or adopt secure, in-house AIA models to handle sensitive datasets. These models must be cost-effective, align with global best practices in AIA, and be adaptable to different fishery boundaries or ecosystems. Hosted on secure agency servers, such systems can ensure confidentiality while enabling the appropriate aggregation of commercial data for public release to meet both fishery and agency EBFM needs, whilst also being standardised to contribute to the broader scientific community [38] to support marine EBM and marine spatial planning.

**Fig 1:**
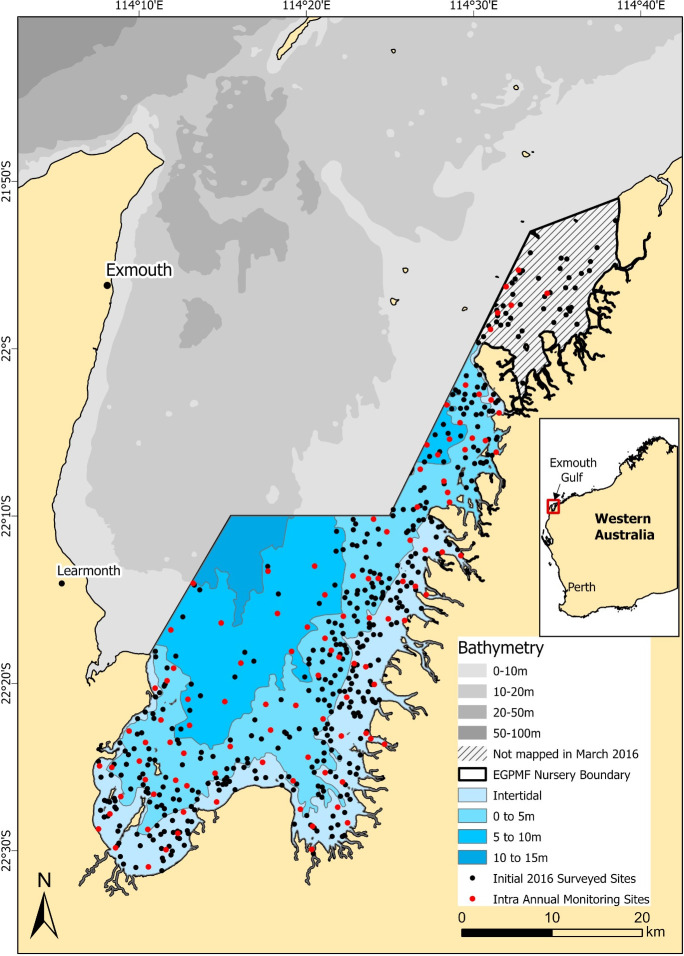
Map of Exmouth Gulf with imagery collection locations between 2016 and 2019. The black dots demonstrate the initial 2016 survey site locations and the red dots indicate the sites monitored intra annually (summer and winter) between 2017 and 2019.

This study focuses on the Exmouth Gulf Prawn Managed Fishery (EGPMF) nursery grounds, which is a specialised fishery management zone that encompasses ~1,139km^2^ of shallow (<20m), mostly turbid waters in southern and eastern extents of Exmouth Gulf in the remote tropical arid Gascoyne coast of Western Australia (22°0’S, 114°20’E) (Fig 1) [39,40]. The EGPMF has historically recorded recruitment failures, which have been linked, in part, to the likely loss of critical habitats in the nursery grounds from large scale natural perturbations (e.g., cyclones) [39,41]. While the relationship between habitat loss and recruitment failure were not quantified, due to the lack of suitable of baseline habitat data, the known stock-recruitment relationships in the EGPMF [39,42,43] demonstrates the need for the development of a rapid, cost effective habitat assessment and monitoring program which can correlate to EGPMF recruitment models [44]. With the time between broodstock surveys and recruitment surveys for the EGPMF being approximately six months [39,43,44], the use of a robust and accurate AIA model to expedite processing of fisheries habitat data is critical for the delivery of a habitat assessment and monitoring program for this fishery.

In this study, we develop and compare two AIA models to evaluate their ability to classify the benthic habitats of Exmouth Gulf. The first model, a grid-based annotation model, replicates the manual annotation method previously undertaken within the study area by generating labels at gridded points. The second, an image-level object detection model, provides a list of which types of objects are present within an image without attempting to match a grid-based survey. This study aims to compare the accuracy, precision, and suitability of the two techniques for fisheries habitat assessments, evaluating the trade-offs between spatial resolution and generalisation. Although this study does not quantify biological relationships between benthic habitats and fish and fisheries productivity, the aim of developing suitable AIA models is to support future analyses by enabling rapid, broad-scale classification of key habitat types from commercial imagery, allowing managers to prioritise ecologically relevant datasets for further investigation. By leveraging widely adopted algorithms and robust fisheries datasets, our methodology aims to address the challenges faced by fisheries management agencies to provide rapid, cost effective first pass habitat classifications using commercial data to support and integrate towards EBFM and broader multi-user marine spatial management [45].

## Methods

### Data collection and manual annotation

**Fig 2:**
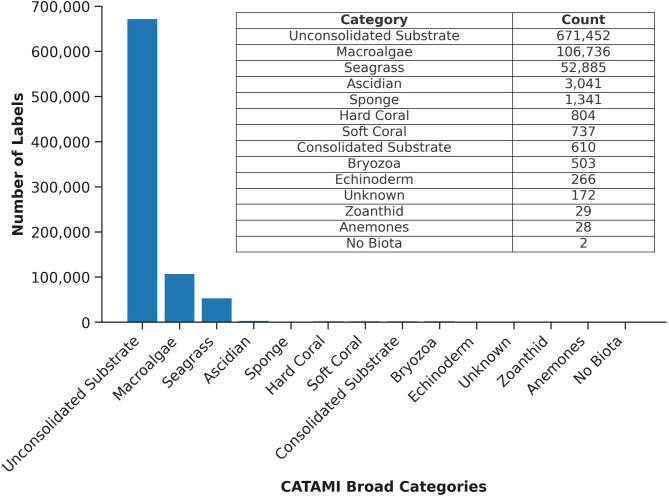
Distribution of manually annotated CATAMI-broad habitat labels for AIA training and testing. Inset table displays the number of labels assigned to each habitat type prior to consolidation into four classes*.*

Annotated imagery data for testing and training the AIA models were derived from imagery of benthic habitats collected during six separate sampling trips between the March 2016 and March 2019. Imagery was collected using a tethered drop video system equipped with a geo-referenced GoPro Hero3/3 + camera mounted on a drop lander, capturing a 0.2 m² area of benthic imagery per drop. At each site, ten static frames were captured along 50 m transects, with drops occurring every 3–7 m, resulting in 2 m² of imagery per site. Habitat surveys in 2016 sampled up to 455 sites in summer and 539 sites in winter, while subsequent monitoring (2017–2019) revisited up to 103 sites per season from a subset of the original survey locations (Fig 1). Collectively, sampling yielded a total of 13,128 benthic images. Each image was processed using the manual annotation software TransectMeasure (www.seagis.com.au), with trained analysts annotating an 8 x 8 grid per image, generating 640 annotated points per site (Fig 2). Habitat features were manually labelled to the highest resolution of the collaborative and automated tools for analysis of marine imagery (CATAMI) classification scheme [46], however, only the broad-level CATAMI categories were used for AIA model development. This comprehensive manually annotated dataset provided a robust foundation for training and validating AIA models, to efficiently classify key habitat types in the EGPMF nursery grounds.

All model training and evaluation used only the extensive manually annotated dataset, no model-generated predictions were used to retrain or refine either model. Although imagery was collected across multiple seasons and years (2016–2019), potential ecological succession or temporal trends in community composition were not explicitly analysed. However, by training the models on this multi-year dataset, we aim to capture natural spatial and temporal variability in benthic habitats to improve model outputs. All field data used in this study were collected under an Instrument of Exemption issued pursuant to the *Fisheries Resource Management Act 1994* (Western Australia), by the Department of Primary Industries and Regional Development, Western Australia, which authorised sampling for fisheries research purposes in the study area.

The manually annotated dataset used in our AIA model analysis exhibited a highly imbalanced distribution of labels, where the top three classes (unconsolidated substrate, macroalgae, and seagrass) accounted for 99.1% of all annotations (Fig 2). Due to imbalances in the dataset, ecologically similar labels were consolidated into four broad classes: unconsolidated substrate, macroalgae, seagrass, and reef structure (Fig 2 and Table 1), with remaining categories excluded from the modelling. This approach was considered acceptable to achieve reliable model training while maintaining ecological relevance for EBFM and EBM reporting in the study area.

**Table 1:** List of consolidated classes used to train the AIA model.

Original Label Description	Reduced Label
Unconsolidated Substrate (Sand)	1_UnCon Sub
Macroalgae	2_MA
Seagrass	3_SG
Sponge	ReefStructure
Hard Coral	ReefStructure
Soft Coral	ReefStructure
Consolidated Substrate	ReefStructure

### Grid-based annotation model

**Fig 3:**
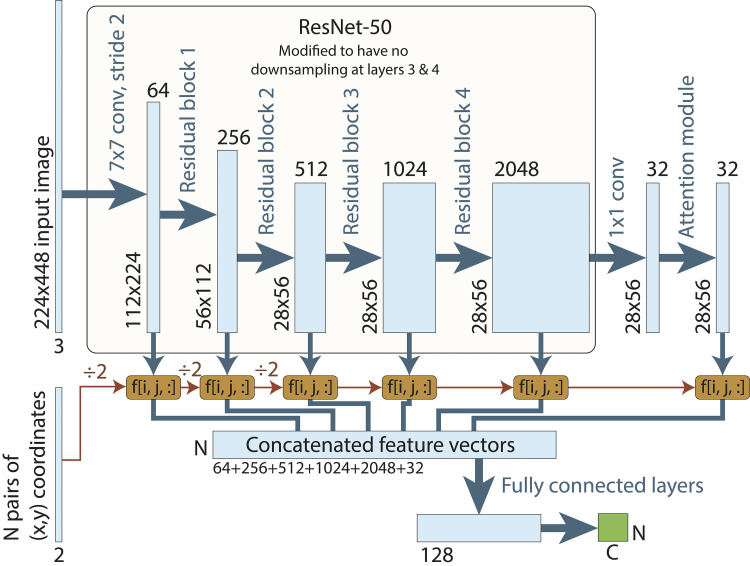
Schematic diagram of the highest-performing neural network architecture for AIA machine learning analysis. Light blue boxes represent tensors at each stage, with spatial dimensions and channel numbers indicated. N denotes the number of coordinate pairs for label prediction, and C represents the number of output classes (typically in this work N = 64, C = 4).

Before training the model, all input images were cropped and resized to 448 × 224 pixels to ensure a standardised resolution for annotation and feature extraction. A modified ResNet-50 framework [47,48] then served as the foundation neural network architecture for the model (Fig 3). The ResNet-50 base model was modified to suit the grid-based annotation, which can be thought of as a sparse version of per-pixel image segmentation. Instead of outputting a segmentation decision for every pixel, the model is provided with a list of pixel coordinates at which a classification output is required. These coordinates are used to sample the ResNet-50 feature vectors at the specific locations that were requested. Since the ResNet-50 model down samples the image between certain blocks, the pixel coordinates were similarly rescaled at each stage. We also modified the ResNet model so that it did not down sample the image between blocks 3 and 4, to retain spatial resolution in the feature vectors. The feature vectors after each residual block were concatenated then analysed using a fully connected neural network (Fig 3).

**Fig 4:**
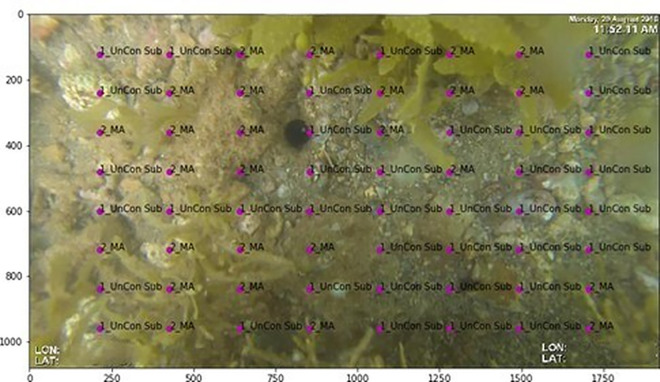
Example of grid-based annotation with 64 predefined points applied to a benthic image. Labels indicate habitat classifications (e.g., Unconsolidated Substrate (UnCon Sub) and Macroalgae (MA)). This structured approach aligns with manual survey methodologies and supports feature extraction for habitat classification.

A grid-based annotation model was implemented to systematically assign labels to 64 predefined points per image, corresponding to a structured 8 x 8 grid which ensured alignment with manual annotation methodologies commonly used in benthic habitat surveys (Fig 4). Each grid point represented a fixed spatial coordinate, ensuring consistent annotation placement across all images. The model extracted features from these locations, indexing feature maps from residual blocks 1, 2, 3, and 4 to capture both fine details and broader spatial patterns. Given the 28 × 56 feature map resolution, each extracted feature corresponded to an 8 × 8 pixel region in the original image, allowing the model to integrate patterns in colour, texture, and structure within a localised patch. These feature vectors were then concatenated before further processing, ensuring that each grid point’s habitat label was based on a combination of spatial information from different network depths.

To further improve classification accuracy, an attention module was incorporated after the final ResNet block. This strategy, derived from its successful application in natural language processing and adapted for vision models [49,50], allowed the network to recognise spatial dependencies and enhance feature discrimination in complex images. Trials were conducted to evaluate various configurations of the attention module, testing its placement after the third, fourth, or both, residual blocks. Performance was found to be comparable between the configuration with two attention modules and one placed solely after the fourth block. For computational efficiency, the single fourth block set up was adopted. Additionally, reducing the down sampling from 2 to 1 in residual blocks 3 and 4 [51], slightly improved performance by preserving more spatial resolution. Introducing dilation to these convolutional elements had minimal impact.

The manually annotated dataset was then randomly split into training (80%) and testing (20%) sets, ensuring the testing set remained unseen and was only used exclusively to evaluate model performance. For each grid point in the testing set, the predicted labels were compared with the manually assigned labels to calculate key performance metrics, including accuracy, precision, and recall. A confusion matrix was used to summarise the model’s ability to classify each habitat class by tabulating the counts of true positives (correct classifications), false positives (incorrect classifications as the target class), true negatives (correctly classified as not belonging to the target class), and false negatives (missed classifications of the target class). Metrics such as precision (proportion of predicted positives that are correct) and recall (proportion of actual positives correctly identified) provided insights into the model’s strengths and weaknesses for each habitat class. During training, data augmentation was implemented by the fastai library [52] with default settings plus vertical flipping to increase robustness. Finally, the model was trained to minimise the cross-entropy loss, when averaged across all labels.

### Image-level object detection model

The same dataset of manually annotated benthic imagery used for the grid-based annotation model was also used for developing the image-level object detection model, randomly split into 80% training and 20% testing. Unlike the grid-based approach, which assigns labels to specific spatial points, this model identifies the presence of the habitat types within different regions of an image. This makes the model computationally simpler, as the system only identifies the presence of habitat classes within the patches, without quantifying their amount or location. Although this approach may omit minor or sparse features (e.g., thin blades of seagrass), its error rate is hypothesised to be comparable to grid-based surveys.

**Fig 5:**
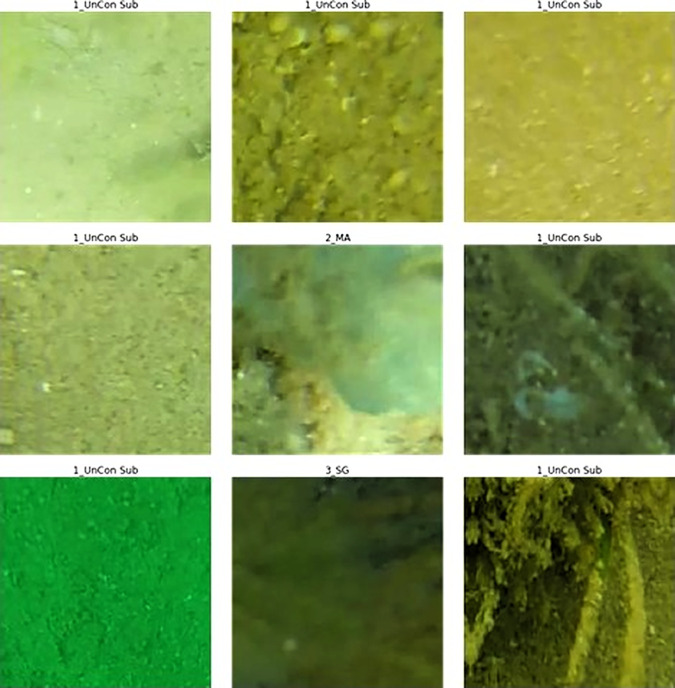
Example of small image patches extracted from full images, (e.g., Fig 4) around an individual labelled point, highlighting modelling limitations at this resolution, such as over-zooming, loss of spatial context, and multiple habitat types within a single patch.

**Fig 6:**
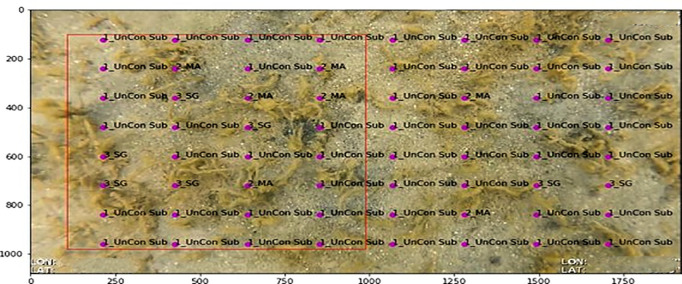
Example of a larger image extraction patch (red box), showing improved spatial context compared to small patches. Each source image was divided into three overlapping patches (left, centre, and right) to capture multiple habitat types and reduce misclassification errors. Shown here is a left patch.

Initially, small patches (224 x 224 pixels) of the images were extracted, centred around each of the labelled points (Fig 5), following previous reported approaches (Mahmood et al., 2016, 2020). However, the resultant patches where often zoomed in too far, making it difficult to distinguish features, or the patches contained multiple objects which, because of the point based precision of the manual annotated dataset, would lead to misclassification when a single label was assigned (Fig 5). To address these limitations, larger patches (880 x 880 pixels) were extracted, covering multiple labelled points within a single region (Fig 6). Each image was divided into three overlapping patches (left, centre and right), with each patch assigned a binary label indicating the presence or absence of all four habitat classes. For example, the patch shown in Fig 6 was labelled as 1_UnCon Sub = True, 2_MA = True, 3_SG = True, and ReefStructure = False. This approach aimed to provide more spatial context, allowing the model to account for the coexistence of multiple habitat types, rather than focus on a single point (Fig 6).

The large-patch image-level object detection model was implemented using a pretrained ResNet-101 network [7], followed by two fully connected layers acting as classifiers. The model processes image patches through ResNet-101, which extracts patterns and spatial structures relevant to habitat classification. These extracted features are then passed to a hidden layer with 512 units followed by a linear output layer producing four binary outputs, corresponding to the presence or absence of the four habitat classes. The large patch image-level model was initially trained with the three patches (left, centre and right) per image, each resized to 224 x 224 pixels, and subsequently fine-tuned to larger inputs of 448 x 448 pixels to capture more detailed spatial information. Standard data augmentation techniques, including random cropping, rotation, and vertical flipping, were applied during training to enhance model robustness. The model was optimised using binary cross-entropy loss for each class, ensuring equal emphasis on all four habitat types. Model performance was evaluated by comparing the predicted presence or absence of each habitat class within a patch to the aggregated manual annotations of labelled points within the same patch. Accuracy, precision, and recall were calculated for each class to assess classification performance, based on the 80% training and 20% testing datasets.

## Results

The performance of the AIA models developed for this study, using imagery of benthic habitat within the EGPMF nursery grounds, demonstrated a high overall accuracy with 90.8% of labels assigned by the grid-based annotation model (Table 2), and 92.9% for the image-level object detection model (Table 3). However, differences emerged in how each model classified specific habitat types and their potential applications. The grid-based model provided more spatially precise classifications, while the image-level model leveraged broader spatial context, simplifying classification by not requiring point-based labels. 

**Table 2:** Performance metrics of the grid-based annotation model.

Metric	Result			
**Overall Accuracy**	90.8%			
	**Unconsolidated Substrate (e.g., sand)**	**Macroalgae**	**Seagrass**	**Reef Structure**
**Precision**	96.4%	73.3%	59.6%	45.5%
**Recall**	94.2%	76.7%	72.2%	68.7%

**Table 3:** Performance metrics of the image-level object detection model.

Metric	Result			
**Overall Accuracy**	92.9%			
**Patch-wise Accuracy** (proportion of patches for which the presence or absence of all four classes is correct)	74.2%			
	**Unconsolidated Substrate**	**Macroalgae**	**Seagrass**	**Reef Structure**
**Precision**	99.6%	85.0%	80.7%	36.8%
**Recall**	99.2%	83.0%	86.7%	65.0%

### Performance of grid-based annotation model

**Fig 7:**
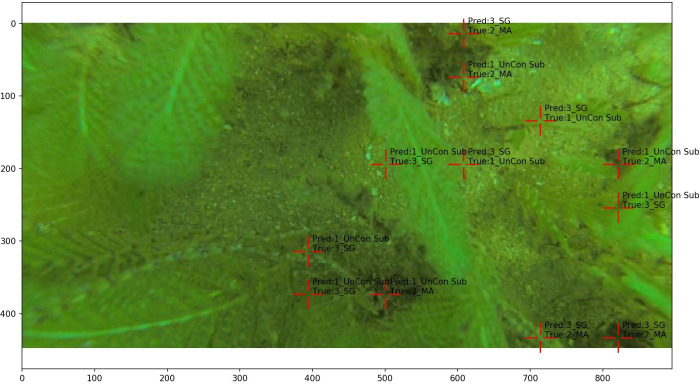
Example of classification errors due to ambiguous grid point placement, particularly at habitat boundaries (e.g., between seagrass and sand). The model’s predicted labels (Pred) are compared to the manually annotated labels (True), highlighting common misclassifications.

The grid-based annotation model achieved an overall accuracy of 90.8%, with precision and recall varied across habitat classes (Table 2). Unconsolidated substrate exhibited the highest precision (96.4%) and recall (94.2%), reflecting the model’s strong performance for this dominant and visually distinct class. Macroalgae demonstrated moderate precision (73.3%) and recall (76.7%), indicating reliable identification of this habitat type, although the moderate precision suggests occasional false positives where visually similar habitats might be misclassified as macroalgae. Seagrass exhibited a precision of 59.6% and recall of 72.2%, indicating the model is relatively effective at detecting seagrass (reasonable recall) but generates more false positives compared to other classes. The imbalance between precision and recall suggests the model occasionally overpredicts the seagrass class, likely due to the upright, thin morphology of some species, such as *Syringodium isoetifolium,* particularly at habitat boundaries (Fig 7). Reef structure exhibited the lowest precision (45.5%) and recall (68.7%), likely due to its limited representation in the training dataset (Fig 2), the high diversity within this class due to category consolidation, and the inherent complexity of detecting smaller or less distinct features. The low precision indicates a higher rate of false positives, suggesting the model overpredicts reef structure by misclassifying other habitats as reef.

These results highlight the model’s dependence on sufficient and balanced training data to maintain high classification accuracy. Errors in classification may stem from an imbalance in the dataset, particularly the underrepresentation of reef structure, which limits the model’s ability to distinguish this class. Additionally, imbalances between precision and recall, particularly for seagrass, suggest overprediction due to habitat similarities. Other sources of misclassification include ambiguity at habitat boundaries, habitat complexity (e.g., epiphytic turf algae growth on seagrass), and potential inaccuracies within the training dataset due to human annotation error.

### Performance of image-level object detection model

The image-level object detection model achieved an overall accuracy of 92.9%, indicating strong performance in classifying the benthic habitats within the EGPMF nursery grounds (Table 3). With a patch-wise accuracy of 74.2%, the model also effectively identified the presence or absence of all four habitat classes within each 448 × 448 pixel patch. These results highlight the model’s ability to leverage broader spatial context by analysing larger image patches, simplifying the classification task by not requiring spatially precise labelling. Performance metrics varied across habitat classes (Table 3). Unconsolidated substrate exhibited the highest precision (99.6%) and recall (99.2%), indicating strong reliability for this dominant class. Macroalgae and seagrass also demonstrated robust precision (85.0% and 80.7%, respectively) and recall (83.0% and 86.7%, respectively), reflecting the model’s effectiveness in detecting these habitats. As with the grid-based annotation model, the limited representation of reef structure, resulted in low precision (36.8%) and recall (65.0%) for this class.

The high recall values observed in the image-level object detection model indicate strong potential for filtering large unannotated datasets and identifying frames of interest in video data for manual inspection. Compared to the grid-based model, which provides spatially precise classifications, the image-level model simplifies classification by analysing broader spatial areas, reducing the reliance on point-based annotations. This model effectively identifies dominant habitat types but is less precise for underrepresented classes, such as reef structure. The ability to screen complex or diverse imagery suggests its utility in prioritising datasets that require further manual review, improving efficiency in large-scale habitat classification.

## Discussion

In this study we developed, evaluated and presented two residual network-based AIA models, both of which observed an overall accuracy greater than 90%. The models were designed to evaluate whether bespoke in-house models could be used to process habitat data, simultaneously supporting the EBFM requirements of the Western Australian fisheries management agency (DPIRD) and managing real or perceived confidentiality concerns when using data from commercial collection sources. Leveraging a large fisheries dataset of benthic imagery from the EGPMF nursery grounds (Fig 1), which was manually annotated by trained analysts, we adapted and expanded upon established machine learning frameworks to create both an in-house grid-based annotation model [47] and an in-house image-level object detection model [53,54]. Both models demonstrated high overall accuracy in classifying benthic habitat, with consistently strong precision and recall values, particularly for the dominant habitats in the EGPMF nursery grounds (i.e., unconsolidated substrate, macroalgae and seagrass) (Fig 2). These results demonstrated the model’s ability to effectively predict the occurrence of these habitats in the image database, in line with human expert manual annotation. While our study uses a unique manually annotated fishery image dataset, the strong predictive performance and accuracies of our models closely aligns with results from similar studies in benthic habitat classification, which demonstrated comparable accuracy to human expert annotations [21,25,26] and achieved similar precision and recall values for features such as macroalgae [54].

Performance results for both our grid-based annotation model (Table 2) and image-level object detection model (Table 3), align closely with those reported in similar AIA based benthic habitat classification studies [54]. For example, Mahmood et al. (2020) applied hierarchical classification methods using features from deep residual networks (ResNet-50) to classify kelp and other benthic species on the Benthoz-15 dataset. They achieved 90% accuracy for a binary “kelp versus not-kelp” classification, with a precision of 71% and recall of 65% in the flat classification approach. When extended to classify across all 145 classes in the dataset, their accuracy dropped to 57.6%, largely also due to imbalanced class representation. Notwithstanding the limitations of imbalanced datasets, the high overall accuracy, precision, and recall values of both models developed in our study underscore their effectiveness in accurately discriminating dominant habitats in the EGPMF study area.

A key feature of our study is the adaptation of the 8 x 8 grid-based annotation model, which integrates spatial and semantic mapping to achieve finer scale habitat classifications. Whole-image classification approaches commonly used in computer vision [19,55–57] can oversimplify diversity and abundance, while pixel-wise image segmentation [51] requires time-consuming labelling to annotate the precise boundaries between habitat classes. The 8 x 8 grid-based labelling scheme used in our study balances spatial precision with sufficient imagery to provide adequate semantic information to accurately describe habitats, mirroring the process used by human analysts, as seen by the achieved 90.8% overall accuracy and precision and recall values of between 59.6% and 96.4% for the dominant key benthic habitats of the EGPMF nursery grounds. Our grid-based model also bridges the gap between manual annotation and automated classification by associating labels with precise spatial locations. This structure supports existing long term fisheries habitat monitoring programs that use percentage composition and density estimates [58], while achieving comparable accuracy to the image-level detection model and other optimised AIA approaches [21,25,26]. As with other AIA models, dataset imbalances hindered model performance of underrepresented categories [59], such as reef structure. However, performance was strong in classifying dominant habitat types, even when distinguishing ambiguous features such as fragmented seagrasses, which are common in the EGPMF nursery grounds [60,61]. While improvements in dataset balance and additional training data would likely enhance classification performance, the grid-based model offers a scalable and spatially precise approach for fisheries habitat assessments, supporting EBFM and broader marine spatial planning.

The AIA models investigated here show the validity of the proposed approach of developing in-house models for habitat analysis. However, a key challenge is the reliance on large manually annotated, fishery-specific training datasets, which may affect the ease of scalability, adaptability and accuracy of the in-house models to other fisheries or regions [12–14]. Addressing this issue would require augmenting fisheries datasets with external data sources, such as FathomNet [62], which although providing a standardised labelled data set, may still presents compatibility challenges related to labelling frameworks, annotation standards, and classification schemes. Adapting datasets can be resource-intensive in the short term and may also result in duplication of effort with existing externally hosted platforms (e.g., www.reefcloud.ai; www.coralnet.ucsd.edu) [21,25,26]. However, while externally hosted platforms provide valuable infrastructure for large-scale annotation and model training, they typically adopt pre-defined classification schemes that may not align with spatially explicit labelling approaches used in fisheries specific applications. Further, they don’t address the challenge of many fisheries agencies in the handling of confidential or proprietary data [33–35]. Therefore, while developing in-house fisheries specific AIA models requires a level of upfront staff resources for manual annotation, and sufficient computational infrastructure, these upfront costs may be offset by long-term benefits in terms of data security, stakeholder trust, and operational efficiency [36,37]. Furthermore, because most of the data analysis would be undertaken by the tailored in-house AIA model, with some retention for data validation by trained human analysts to review uncertain or ambiguous outputs [63], future savings in resourcing can be used to improve, expand or refine data collection. To enhance transferability, future research should explore methods to aggregate data to a publicly available level, enabling the sharing of standardised training datasets across agencies while maintaining data security.

By leveraging established machine learning models, grounded in the principles of global best practices [19,21,26,38,47,54], and adapting them for in-house use by applied fisheries scientists, our study addresses a unique challenge faced by fisheries management agencies in adopting artificial intelligence use for processing potentially commercially sensitive fisheries data [34,37]. In doing so, we provide greater opportunity for fisheries, and fisheries resource managers, to further incorporate fishery-specific datasets, particularly in relation to collected benthic habitat imagery, to support sustainable EBFM and broader marine spatial planning [64,65]. Specifically, the models developed in our studies, demonstrate high accuracy in identifying key habitats for penaeid prawn recruits, such as seagrass and macroalgae [40,41,60]. These models will further support the development of near real time habitat assessment and monitoring for the EGPMF, using benthic image data collected from the nursery grounds, further supporting EBMF and good industry stewardship of a marine environment facing cumulative natural and anthropogenic pressures [45,66]. However, it is important to emphasise that AIA represents just one tool in a broader suite of habitat assessment and monitoring techniques, each with different strengths, limitations, and data resolutions [67]. Within an EBFM framework, effective advice relies not only on the availability of data, but an understanding of the confidence and constraints of each method used [30], whether from automated imagery, direct sampling, or integrated approaches [68,69]. Future research should prioritise the validation of these models in other similar fisheries management scenarios, such as Shark Bay and the Shark Bay Prawn Managed Fishery, and assess their effectiveness, compared to other survey methods, in supporting decision-making. A key area of investigation is their ability to detect temporal variations in habitat, which is critical for adaptive fisheries management and long-term ecosystem monitoring.
